# Interaction of Aqueous Bovine Serum Albumin with Silica Aerogel Microparticles: Sorption Induced Aggregation

**DOI:** 10.3390/ijms23052816

**Published:** 2022-03-04

**Authors:** Attila Forgács, Madalina Ranga, István Fábián, József Kalmár

**Affiliations:** MTA-DE Redox and Homogeneous Catalytic Reaction Mechanisms Research Group, Department of Inorganic and Analytical Chemistry, University of Debrecen, Egyetem tér 1, H-4032 Debrecen, Hungary; forgacs.attila@science.unideb.hu (A.F.); madalina.ranga@mail.ru (M.R.); ifabian@science.unideb.hu (I.F.)

**Keywords:** aerogel, sorption, serum albumin, reaction kinetics, mechanism

## Abstract

Mesoporous silica aerogels have a wide range of potential applications in biotechnology, the food industry, pharmacy and medicine. Understanding the nature of the interactions of biomolecules with these porous nanostructured materials is essential for achieving optimum performance in the targeted applications. In this study, the well-characterized bovine serum albumin (BSA) was chosen as a model protein to probe protein–aerogel interactions in the solution phase. Aqueous BSA was mixed with suspended silica aerogel microparticles, and the colloid system was monitored on-line by UV–vis spectrophotometry and turbidimetry. The global mathematical analysis of the time-resolved data reveals that the fast sorption of the protein on the aerogel microparticles follows a multistep binding mechanism. The extensive sorption of the protein eventually induces the aggregation of the covered aerogel due to the alteration of the electrical double layer of the particles. The interaction of BSA and silica aerogel is the strongest between pH = 4 and 5, because their native surface charges are the opposite in this pH range, as indicated by their respective zeta potentials.

## 1. Introduction

Mesoporous silica aerogels are known for their wide range of potential applications in biotechnology and medicine, mainly as enzyme and drug carriers and tissue scaffolds [1,2,3,4,5]. The advanced applications generate increasing demand for understanding the nature of the interactions of these nanostructured materials with biomolecules. This is essential for increasing their effectiveness and assessing their safety [6]. The primary physico-chemical phenomena governing such interactions are the reversible or irreversible sorption of the biomolecules on the accessible silica aerogel surface [7]. The increasing demand for nanostructured materials in biomedicine- and biotechnology-related applications resulted in the development of advanced aerogels, such as cross-linked polysaccharides, hybrids and composites that outperform the archetypical silica [2,3]. However, for understanding the fundamental principles of biomolecule–aerogel interactions, the well-characterized silica aerogel is a reasonable choice as a representative model material.

Aerogels are highly porous and consist of an open-cell pore network. Their main characteristics are high specific surface area (500–1200 m^2^ g^−1^), high porosity (80–99.8%), low density (∼0.003–0.5 g cm^−3^), low thermal conductivity (0.005–0.1 W/mK), ultra-low dielectric constant (*k* = 1.0–2.0) and low refraction index (∼1.05). Silica-based aerogels are biocompatible as shown by an ever-growing number of in vitro and in vivo studies [6,8,9]. This makes them promising candidates for enzyme immobilization supports, drug delivery vehicles and bone regeneration implants.

Serum albumins are widely used model proteins in research. Bovine serum albumin (BSA) is utilized as a basis of drug delivery devices, biocatalysts and enzyme inhibitors [10,11,12]. BSA is a soft protein; therefore, its aqueous tertiary structure is significantly influenced by the pH of the solution [11,13]. This feature is fundamental in understanding its properties for binding to solid surfaces and transport [14]. The mechanisms of BSA sorption on various silica surfaces have been studied in the pH range between 3 and 9 [15,16,17]. The protein starts to unfold only at pH values lower than 4 and higher than 9. BSA first forms protein islands on a silica surface and, finally, completes the monolayer. Multilayer sorption and irreversible binding have also been observed. The structure of the adsorbed protein remains highly intact only in a narrow pH range around pH = 4.7.

Protein sorption and binding on solid surfaces are complex processes that are sensitive to the physico-chemical environment (temperature, pH, ionic strength, etc.), as well as to the chemical characteristics and the morphology of the sorbent [16,18]. Several authors have proposed a two-state model for the sorption of proteins on solid surfaces resulting in weaker (reversible) or stronger (irreversible) binding modes. There are two general mechanisms proposed for the two-state binding of proteins: (1) A transition pathway where all proteins initially adsorb by weak interactions, but some subsequently undergo a transformation into strong binding. (2) Parallel sorption of the proteins resulting in either a weakly bound state or directly forming a strongly bound state [7,19,20,21]. Turbidimetry and UV–vis measurements have been shown to be fundamental in quantifying the interactions of proteins with surfaces including sorption and aggregation [22,23].

In this paper, the interaction of aqueous BSA and suspended silica aerogel microparticles was investigated by time-resolved experiments. Archetypical mesoporous silica aerogel was used. The dissolved protein was injected into the aerogel suspension, and subsequent changes in the colloid system were monitored on-line by UV–vis spectrophotometry and turbidimetry. The time-resolved data were evaluated by global kinetic analysis and a detailed mechanistic model is postulated. The pH-dependent interaction between BSA and the aerogel is elucidated based on zeta-potential (ζ) data.

## 2. Results and Discussion

### 2.1. Morphology of the Silica Aerogel

**Figure 1:**
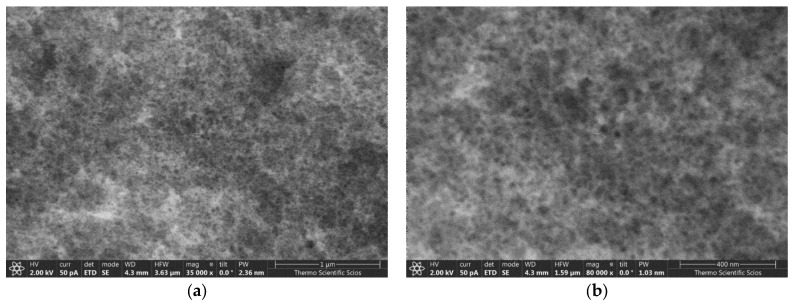
Low-voltage scanning electron microscopy (LV-SEM) images of pristine mesoporous silica aerogel at 35k× (**a**) and 80k× (**b**) magnifications. The scale bars are 1 µm (**a**) and 400 nm (**b**).

**Figure 2:**
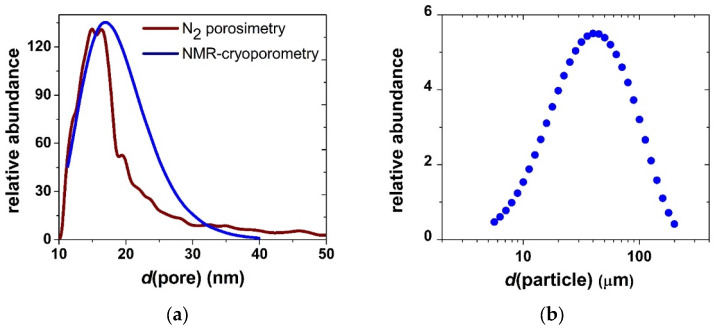
(**a**) Pore size distribution of silica aerogel in the dry material and in the suspended microparticles measured by N_2_-sorption porosimetry and NMR cryoporometry, respectively. (**b**) Size distribution of suspended silica aerogel microparticles measured by LDLS.

Representative low-voltage scanning electron microscopy (LV-SEM) images of the pristine silica aerogel are shown in Figure 1. The solid backbone of the material is composed of covalently bound quasi-spherical nanoparticles, defining the frame of the interconnected pore network. The apparent specific surface area (979 m^2^ g^−1^) and the pore size distribution of the pristine aerogel were determined by N_2_-sorption porosimetry (Figure 2a). The mesoporous structure of unimodal pore size distribution is typical for silica aerogel.

When silica aerogel is dispersed in aqueous media, it forms a stable suspension of microparticles. The size distribution of the particles was measured by laser diffraction light scattering (LDLS), and the results are shown in Figure 2b. The pore network of the hydrated aerogel particles was characterized by NMR cryoporometry and diffusiometry. The results and discussion are reported in an earlier publication [24]. The main conclusion is that interconnected highly permeable mesoporous structure of silica aerogel is conserved when the aerogel forms microparticles in water (cf. Figure 2a).

### 2.2. Effect of pH on Zeta Potentials of BSA and the Silica Aerogel

**Figure 3:**
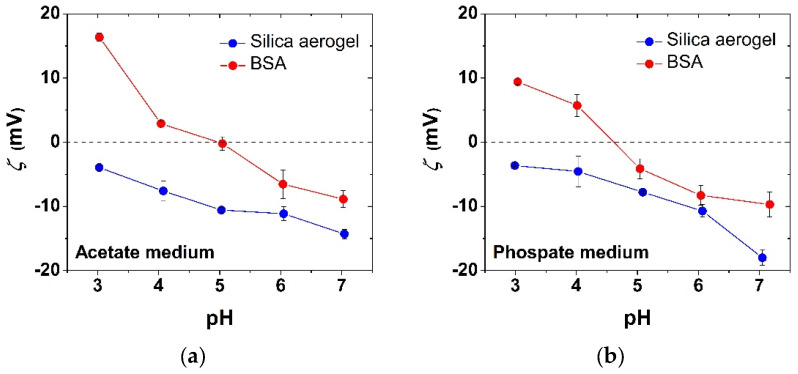
Zeta potentials (ζ) of aqueous BSA and silica aerogel microparticles as a function of pH in the presence of 0.01 M acetate (**a**) or phosphate (**b**) electrolytes at constant ionic strength *I* = 0.10 M.

The ζ-potentials of aqueous BSA and silica aerogel microparticles are shown in Figure 3 as a function of pH in the presence of acetate and phosphate electrolytes at a constant ionic strength of *I* = 0.10 M set by NaCl. The isoelectric point of BSA is between pH = 4.5 and 5.0 in both media, which is in good agreement with the literature [11]. Silica aerogel particles remain negatively charged in the studied pH range regardless of the nature of the electrolyte. The trends in the ζ-potential values as a function of pH are slightly different depending on whether acetate or phosphate is present both for BSA and for silica aerogel, which indicates specific interactions with these ions. Acetate and phosphate anions, depending on their hydrodynamic radii and surface charge densities, bind to the hydrated protein surface in different modes, and alter the electrical double layer.

These ion-specific interactions arise from the Hofmeister series, which is well established in protein systems [25,26,27]. The fact that the ζ-potential of silica aerogel is negative even at pH = 3.0 strengthens the idea of the specific sorption of phosphate and acetate anions form the corresponding media. In the absence of these ions, the isoelectric point of amorphous silica is reported to be at pH = 3.5 [28].

### 2.3. The Interaction of BSA with Silica Aerogel Microparticles

**Figure 4:**
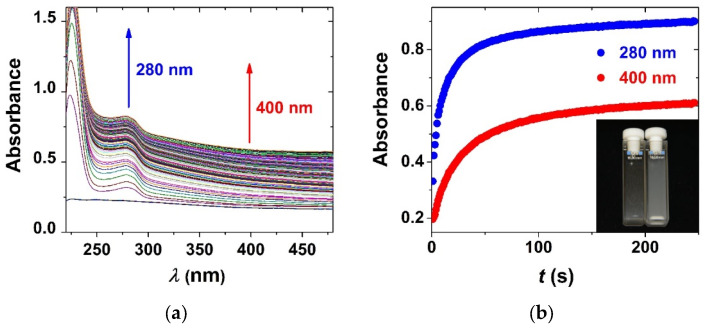
(**a**) Time-resolved UV–vis spectral change following the addition of aqueous BSA to silica aerogel suspension. (**b**) The corresponding kinetic curves at 280 and 400 nm. Inset: photograph of the aerogel suspension in a cuvette before adding BSA and 5 min after mixing. *c*_0_(aero) = 100 μg/mL; *c*_0_(BSA) = 300 μg/mL; pH = 4.6 acetate buffer; 800 rpm stirring; 25.0 °C.

**Figure 5:**
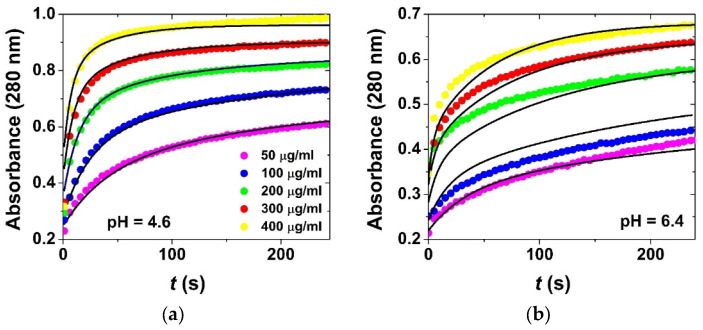
Series of kinetic experiments using different initial concentrations of BSA at pH = 4.6 in acetate buffer (**a**) and at pH = 6.4 in phosphate buffer (**b**). The markers are experimental data points, and the continuous lines are the results of global kinetic data fitting using the model in Scheme 1. Corresponding simulated time-dependent concentration profiles are shown in Figure 6. *c*_0_(aero) = 100 μg/mL; *c*_0_(BSA) = 50–400 μg/mL; 800 rpm stirring; 25.0 °C.

When aqueous BSA is injected into silica aerogel suspension, a rapid increase in turbidity is immediately visible. The corresponding spectral change is well defined in the UV–vis traces (Figure 4). In order to study the mechanism of the interaction of BSA and silica aerogel, their initial concentrations were varied in a series of kinetic experiments at pH = 4.6 in acetate buffer and at pH = 6.4 in phosphate buffer (Figure 5). The time-resolved UV–vis spectral data served as the basis of global kinetic analysis. Two kinetic steps can be observed in all experimental curves regardless of the conditions.

**Scheme 1:**
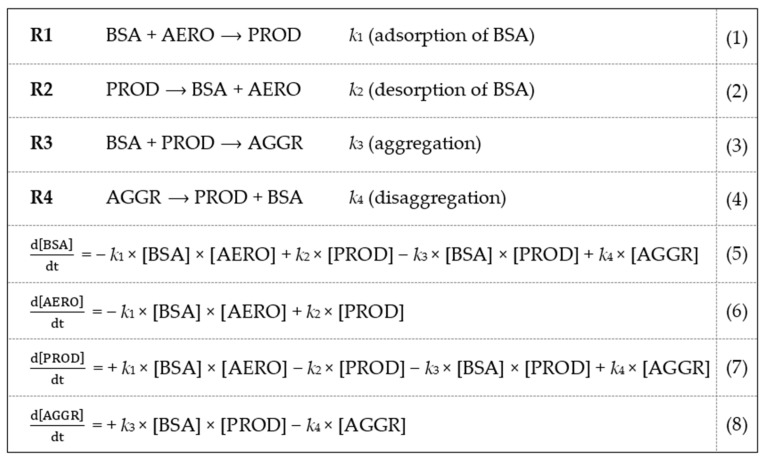
Kinetic model for the sorption of BSA on aerogel particles. Four species are defined: aerogel particles (AERO), bovine serum albumin (BSA), bovine serum covered aerogel (PROD) and aggregate (AGGR). These are interconverted into each other in two reversible processes: R1/R2 and R3/R4. The physico-chemical rationale of the model is given in the text. The *k* parameters are the rate constants. The differential equation system describing the time-dependent concentration of each species ([BSA], [AERO], [PROD], [AGGR]) is given below the reaction steps. Fitted kinetic curves are shown in Figure 5. The estimated kinetic parameters are given in Table 1. Simulated time-dependent concentration profiles are shown in Figure 6.

A simple kinetic model is proposed in Scheme 1 for the interpretation of the physico-chemical changes taking place in the colloid system. The kinetic model consists of two reversible processes (R1/R2 and R3/R4). First, BSA is adsorbed on the aerogel particles (AERO) yielding protein-covered particles (PROD) in R1. Desorption takes place in R2. Second, the subsequent sorption of additional BSA results in the aggregation of the covered aerogel particles (AGGR) in R3. The physico-chemical rationale is that the extensive sorption of BSA alters the electrical double layer of the aerogel particles and, thus, destabilizes the colloid system [17]. Disaggregation takes place in R4. Similar models proposing two stages of protein binding have successfully been used earlier to describe the interaction of BSA with different silica surfaces [7,17]. In the published models, the first step is described as the rapid reversible interaction (adsorption) of BSA with the silica surface, which may be followed by a secondary stronger attachment. The stronger attachment significantly alters the electrical double layer of the silica surface.

The kinetic model in Scheme 1 was used for global data fitting. The time-dependent concentrations of the different chemical species ([BSA], [AERO], [PROD], [AGGR]) were calculated directly from the time-resolved UV–vis spectra on the basis of the following considerations.

A general observation is that the baseline of the recorded UV–vis spectra is elevated in the full studied wavelength range, because of the extinction (apparent absorbance) of the aerogel particles. The light absorption (absorbance) of BSA is superimposed on this baseline (cf. Figure 3). Independent experiments confirm that the measured apparent absorbance of the aerogel particles is directly proportional to their concentrations. This forms the basis of the turbidimetric quantification of the colloid aerogel particles, as demonstrated in several cases in prior literature [22,24,29,30]. Furthermore, Beer’s law is valid for the BSA, i.e., its absorbance is directly proportional to its concentration. Finally, the apparent absorbance of the aerogel particles and the absorbance of BSA are additive. Therefore, the total measured absorbance can be expressed as their sum in every wavelength as given in Equations (9) and (10). The validity of these considerations has been demonstrated in several prior cases dealing with sorption in colloid systems [22,24,29,30].

*A*^t^ = *A*^t^_BSA_ + *A*^t^_AERO_ + *A*^t^_PROD_ + *A*^t^_AGGR_ (9)

*A*^t^ = {ε_BSA_[BSA] + ε_AERO_[AERO] + ε_PROD_[PROD] + ε_AGGR_[AGGR]}*l* (10)

In these equations, *A*^t^ is the time-dependent measured absorbance and the *A*^t^_SPECIES_ are the absorbances of BSA and the different aerogel particles. In Equation (10), the absorbances are expressed for each species as the products of their molar absorbances or extinction coefficients (ε_SPECIES_) and their time-dependent concentrations (*l* is the optical length). The molar absorbance of BSA and the extinction coefficient of AERO were determined at several wavelengths using dilution series of solutions and suspensions in independent calibration experiments both in acetate buffer and in phosphate buffer.

**Figure 6:**
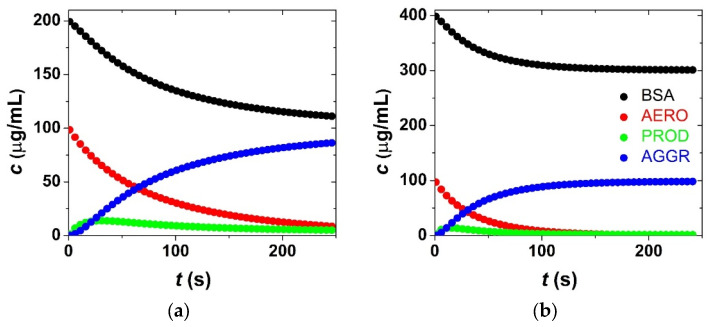
Simulated time-dependent concentrations of aerogel particles (AERO), bovine serum albumin (BSA), bovine-serum-covered aerogel (PROD) and aggregate (AGGR) calculated at two different initial BSA concentrations of *c*_0_(BSA) = 200 μg/mL (**a**) and *c*_0_(BSA) = 400 μg/mL (**b**) at pH = 4.6 in acetate buffer (cf. Figure 5a; *c*_0_(aero) = 100 μg/mL; 800 rpm stirring; 25.0 °C). The simulation is based on the kinetic model in Scheme 1 using the estimated parameters in Table 1. The color code is given in the legend.

For global kinetic data fitting, the differential equations in Scheme 1 were solved numerically and the time-dependent concentrations [BSA], [AERO], [PROD], [AGGR] were substituted into Equation (10). This expression was fitted to the experimental kinetic traces at multiple wavelengths. The final BSA concentrations after the establishment of the equilibrium in the colloid system were determined experimentally by analyzing the clear supernatants. These equilibrium BSA concentrations were used as fixed input data in the global fitting. Additional fixed input data were the molar absorbance of BSA and the extinction coefficient of AERO. All other parameters were estimated by simultaneously fitting all experimental kinetic curves at two wavelengths (280 and 400 nm) using the GEAR algorithm with the ZiTa software [31,32]. The goodness of fit is exceptional in the case of the experiments in acetate medium. The fitted kinetic curves are shown in Figure 5 and the estimated kinetic parameters are given in Table 1. Simulated time-dependent concentration profiles of [BSA], [AERO], [PROD], [AGGR] are shown in Figure 6. Overall, the proposed kinetic model is proved to be adequate to describe the interaction of aqueous BSA with silica aerogel microparticles.

**Figure 7:**
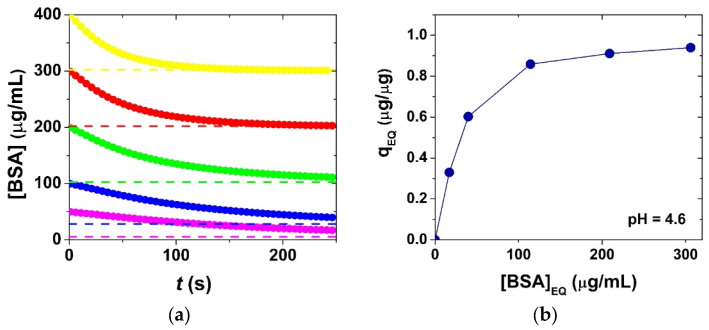
(**a**) Time-dependent concentration of bovine serum albumin ([BSA]) in experiments using different initial BSA concentrations (*c*_0_(BSA) = 50, 100, 200, 300, 400 μg/mL) at pH = 4.6 in acetate buffer (cf. Figure 5a; *c*_0_(aero) = 100 μg/mL; 800 rpm stirring; 25.0 °C). The simulation is based on the kinetic model in Scheme 1 using the estimated parameters in Table 1. (**b**) Sorption isotherm giving the mass of bound BSA per 1 μg of silica aerogel (*q*_EQ_) as a function of the equilibrium concentration of aqueous BSA ([BSA]_EQ_) following the completion of the aggregation process.

The sorption isotherm determined in acetate buffer is shown in Figure 7. This isotherm was calculated from the equilibrium BSA concentrations following the completion of the aggregation process. The classical equilibrium sorption isotherm models (Langmuir, Temkin, etc.) are naturally not adequate to describe the equilibrium state in a two-stage sorption-induced aggregation process, because of the fundamental differences in the underlying mechanisms [7,33]. However, the model in Scheme 1 is adequate to give the equilibrium concentrations of the different species and describe the final state of the aerogel–BSA system under different conditions (cf. Figure 6 and Figure 7). The measured maximum sorption capacity is 0.96 ± 0.10 μg BSA per 1 μg of silica aerogel in acetate buffer at pH = 4.6, which is reasonable in the case of multilayer sorption of proteins. The sorption capacity is significantly less, 0.52 ± 0.10 μg BSA per 1 μg of silica aerogel in phosphate buffer at pH = 6.4. The difference is due to the alteration of the surface charges of BSA and silica as a function of pH (cf. Figure 3). The surface charge of BSA is positive and that of silica is negative at pH = 4.6. However, both BSA and silica are negatively charged at pH = 6.4, which results in a significant decrease in the binding capacity. (Further considerations are summarized in the Section 3).

## 3. Conclusions

The global kinetic analysis of time-resolved UV–vis spectrophotometry and turbidimetry data revealed that the interaction of aqueous BSA with suspended silica aerogel microparticles is a multistep physico-chemical process. The first step is the rapid reversible adsorption of BSA on the surface of the nanostructured aerogel particles. This is followed by multilayer sorption of BSA, which alters the electrical double layer of the silica aerogel particles and induces their aggregation, as clearly seen in the increase of the turbidity of the colloid system. Similar multistep binding models have been proposed in earlier literature to describe the interaction of proteins with various silica surfaces [7,17,18].

In their native states, silica aerogel microparticles form a quasi-stable suspension in aqueous acetate or phosphate media in a wide pH range from 3 to 7. The stabilizing coulomb repulsion among the particles is due to the specific sorption of anions on the silica surface resulting in negative surface polarization in the electrical double layer. This is indicated by the negative ζ-potential values of the particles even at pH = 3.0. The extensive sorption of BSA alters the electrical double layer of the silica aerogel particles, which lowers the stabilizing Coulomb repulsion, eventually resulting in the aggregation of the particles. The specific details of the interactions are as follows.

The sorption of BSA is more extensive at pH = 4.6 in acetate buffer in which the native surface charge of BSA is positive and that of the aerogel is negative resulting in a close to zero net surface charge for the protein-covered particles [17]. For the same reason, the BSA-sorption-induced aggregation of silica aerogel microparticles is somewhat more pronounced at pH = 4.6. The structure of the protein layer at pH = 4.6 is proposed to be a densely packed monolayer or even a multilayer. Electrostatic protein–protein repulsion is at a minimum at pH = 4.6, because the isoelectric point of BSA is close to this value. Thus, the effective surface charge of BSA is close to zero at pH = 4.6, which results in the high packing density of the protein on the aerogel surface [7]. The highly compact structure of BSA under these conditions ensures maximum sorption capacity and the formation of a highly packed protein film on the particles. This BSA layer also has an effective charge close to zero, meaning no stabilizing Coulomb repulsion between the covered particles, and eventually leading to their aggregation [17,21].

## 4. Materials and Methods

### 4.1. Chemicals

Bovine serum albumin was purchased from Sigma-Aldrich (Budapest, Hungary) (BSA: Pc. code: 1003004334 A2153-10G; Lot no. SLCD0987; lyophilized powder; purity ≥96% by agarose gel electrophoresis) and was used without further purification. All other chemicals were purchased from Sigma-Aldrich in “trace metal basis” grade and used without further purification. The aqueous solutions were prepared using doubly deionized and ultrafiltered water (ELGA PureLab, ELGA, Budapest, Hungary).

### 4.2. Synthesis and Characterization of the Silica Aerogel

The silica aerogel was synthesized by the classical sol–gel method and drying in supercritical CO_2_. The detailed synthesis is given in our earlier publication [24]. Briefly, two solutions (A and B) were prepared. Solution A was made from TMOS dissolved in methanol. Solution B contained methanol, distilled water and aqueous ammonia solution. The two solutions were mixed under stirring and then poured into a plastic mold. After 24 h, the alcogel was transferred into a perforated mold in methanol. After 24 h, the sample was soaked in a mixture of acetone–methanol for another 24 h, and this step was repeated by increasing the acetone content every day. Finally, the sample was stored in acetone. The gel was dried under supercritical conditions in CO_2_, as described previously [34].

The morphology of the aerogel was studied using low-voltage scanning electron microscopy (LV-SEM) with a ThermoFisher Scientific Scios 2 instrument (ThermoFisher, Waltham, MA, USA). The sample was fixed on vacuum-resistant carbon tape on the sample holder. Because of the low accelerating voltage and small electron beam current, the charging effects of the aerogel sample were practically eliminated, and the fresh fracture surface of the aerogel was imaged without the application of conductive coating [35].

Nitrogen-sorption porosimetry measurements were performed in a Quantachrome Nova 2200e surface area and porosity analyzer (Quantachrome Instruments, Boynton Beach, FL, USA) after the samples were outgassed under vacuum at 100 °C for 8 h. Apparent specific surface area was calculated using the Brunauer–Emmett–Teller (BET) model. Pore size distribution was calculated using the Barret–Joyner–Halenda (BJH) method [36].

The size distribution of the suspended aerogel particles was measured by a Malvern Mastersizer 2000 (Malvern Instruments, Malvern, UK) laser diffraction light scattering (LDLS) instrument. Data were collected with two lasers of different wavelengths in a flow-through cell. The angular scattering intensity data were evaluated by the instrument controlling software. The particle size is reported as the volume equivalent sphere diameter.

The nuclear magnetic resonance (NMR) cryoporometry characterization of the hydrated silica aerogel is detailed in previous publications [24,37]. The conventional conditions and setup were used. Briefly, melting and freezing experiments were performed on a 360 MHz NMR instrument. The Carr–Purcell–Meiboom–Gill (CPMG) spin-echo pulse sequence was applied with a 1.5 ms delay to filter the signal of solid water. Temperature was calibrated on glycol and methanol. Experiments were started with melting, and subsequent freezing and melting cycles were performed. Spin-echo ^1^H spectra were recorded in 0.25 K steps, keeping the sample at constant temperature for 5 min before measurement.

### 4.3. Zeta Potential Measurements

Zeta potential was measured in a MALVERN Zetasizer Nano ZS (Malvern Instruments, Malvern, UK) dynamic light scattering (DLS) instrument equipped with an electrokinetic cell using conventional instrument setup and operation. Aerogel suspensions were made by wet grinding silica aerogel using a Potter-Elvehjem tissue grinder (10 min) and sonication (10 min). ζ-potentials of dissolved BSA and suspended silica aerogel were measured in acetate and phosphate containing aqueous media at 298 ± 1 K. The aqueous media were prepared using 0.010 M acetic acid or H_3_PO_4_, NaOH and NaCl in order to maintain constant ionic strength (*I* = 0.10 M). The BSA concentration was 0.15 mg/mL, and the suspended aerogel was 0.10 mg/mL. The pH of the samples was set to be between 3 and 7 by the addition of NaOH and measured using a 913 pH Meter (laboratory version) of Metrohm (Budapest, Hungary).

### 4.4. Time-Resolved Sorption Experiments

Acetate and phosphate buffers were prepared by dissolving acetic acid or H_3_PO_4_ at a final concentration of 0.01 M and setting the pH using NaOH. All BSA solutions were freshly prepared before the experiments by dissolving the solid crystalline protein in the desired acetate or phosphate buffer without shaking. Silica aerogel suspension was prepared by wet grinding and sonication as described above. The aerogel concentrations were varied between 50 and 800 μg/mL.

UV–vis spectrophotometry and turbidimetry measurements were carried out in an Agilent 8453 diode array instrument equipped with a built-in Peltier temperature control unit and magnetic stirrer (Agilent Technologies, Santa Clara, CA, USA). The wavelength range was 200–600 nm. A standard quartz cuvette of 1.00 cm × 1.00 cm was used for all measurements. All experiments were carried out at constant temperature (25.0 °C) and under constant stirring (800 rpm) using a cuvette-sized magnetic stirring rod. A series of different concentration solutions were used to determine the molar absorbance of BSA in acetate and phosphate buffers in the used wavelength range. A series of aerogel suspensions with different concentrations was used to validate Equation (10) and determine the extinction coefficient corresponding to silica aerogel particles in acetate and phosphate buffers in the used wavelength range. These are treated as standard external calibration series for UV–vis spectrophotometry (BSA) and turbidimetry (aerogel particles). Turbidimetry and UV–vis measurements have been shown to be fundamental in quantifying the interactions of proteins with surfaces including sorption and aggregation [22,23].

For the time-resolved (kinetic) experiments, 1.0 mL of aerogel suspension was placed in the thermostated spectrophotometric cuvette, and stirring and detection was started. To start the process, 1.0 mL of BSA solution was promptly injected into the suspension. The same buffer of the same pH was used in the suspension and in the BSA solution. The concentration of BSA was varied in a series of experiments. All runs were repeated in triplicate. Absorbance change was followed in the 200–600 nm wavelength range for 300 s with the time resolution of 1.0 s. The evaluation of the recorded time-resolved UV–vis spectra is described in Section 2.3. After 10 min of mixing, the suspensions were centrifuged, and the equilibrium concentrations of BSA in the clear supernatants were measured by UV–vis spectrophotometry.

## Figures and Tables

**Table 1:** Kinetic parameters of the model in Scheme 1 determined by global data fitting. The corresponding fitted curves are shown in Figure 5. *c*_0_(aero) = 100 μg/mL; *c*_0_(BSA) = 50–400 μg/mL; 800 rpm stirring; 25.0 °C.

	*k*_1_ [μg^−1^ Ls^−1^]	*k*_2_ [μg^−1^ Ls^−1^]	*k*_3_ [μg^−1^ Ls^−1^]	*k*_4_ [μg^−1^ Ls^−1^]
pH = 4.6 (acetate)	(7.5 ± 0.1) × 10^−5^	(7.2 ± 0.1) × 10^−4^	(3.6 ± 0.1) × 10^−4^	(1.4 ± 0.1) × 10^−3^
pH = 6.4 (phosphate)	(4.4 ± 0.2) × 10^−5^	(8.0 ± 1.0) × 10^−4^	(5.9 ± 0.4) × 10^−4^	(1.4 ± 0.3) × 10^−3^

## Data Availability

The complete sets of data presented in this study are available on request from the corresponding author.
